# The Influence of Empathy and Consumer Forgiveness on the Service Recovery Effect of Online Shopping

**DOI:** 10.3389/fpsyg.2022.842207

**Published:** 2022-03-30

**Authors:** Jiahua Wei, Zhenyu Wang, Zhiping Hou, Yongheng Meng

**Affiliations:** School of Business, Guilin University of Technology, Guilin, China

**Keywords:** online shopping, service recovery, consumers repurchase intention, empathy, consumer forgiveness

## Abstract

The service failure of online shopping has always plagued online stores, but the current academic circles still need to explore the service recovery of online shopping from the perspective of empathy and consumer forgiveness. Based on the service failure cases of real online shopping, this article uses the method of situational experiment to carry out empirical research, discusses the impact mechanism of service recovery effect from the perspective of empathy and consumer forgiveness, and tests the moderating role of online store reputation. The results show that in the online shopping service recovery scenario, empathy has a positive impact on consumer forgiveness, consumer forgiveness has a positive impact on consumer repurchase intention, and consumer forgiveness plays a mediating effect between empathy and consumer repurchase intention. Online store reputation plays a moderating role in the relationship between consumer forgiveness and consumer repurchase intention. The research conclusion of this article will help to expand the application of empathy and consumer forgiveness in the research of service recovery, enrich the theory of online shopping service recovery, improve the effect of online shopping service recovery, and promote the healthy development of online shopping business models.

## Introduction

The interactivity and intangibility of services determine that service failure is inevitable (Du and Fan, 2007). The same is true for the online shopping industry. Although online shopping has become an indispensable part of people’s lives and profoundly changed people’s production and lifestyle, service failure has plagued the online shopping industry. After service failure, consumers are very disappointed and angry and this failure will be exposed through the media such as WeChat and QQ, which will damage the image of online stores, resulting in a significant decline in sales performance or even bankruptcy. It can be said that service failure has become a lingering curse of online stores and makes the operation mode of online shopping service face new challenges.

Both offline consumers and online consumers will feel that the resources exchanged have depreciated when they encounter service failure, resulting in psychological dissatisfaction and complaints, and service recovery is needed to eliminate the negative impact of service failure (Crisafulli and Singh, 2017). The service recovery strategy is the focus of current service failure and service recovery research. In the current online retail service recovery research, the causes of online retail service failure, service recovery types and strategies, customer participation, transfer cost, perceived fairness, and the impact of corporate social responsibility (CSR) on service recovery satisfaction and loyalty are discussed (Assefa, 2014; Siu et al., 2014; Shafiee and Bazargan, 2018; Fang et al., 2019; Yang et al., 2020). In the research of empathy and consumer forgiveness, some scholars believe that consumers driven by empathy consciously weaken negative motives such as alienation and retaliation and enhance positive motives such as tolerance and kindness, so as to help repair damaged relationships (Mccullough et al., 1998). Sun and Sun (2021) also believe that empathy plays an intermediary role in the impact of service recovery strategies on consumers’ willingness to forgive. However, the current academic community still needs to undertake research on the service recovery effect of online shopping services from the perspective of empathy and consumer forgiveness. It is difficult to explain the impact relationship between empathy and consumer forgiveness and between consumer forgiveness and consumer repurchase intention. This is an urgent problem to be solved in the theory and practice of online shopping service recovery.

This study will try to explore and explain the influence mechanism of variables such as empathy and consumer forgiveness on service recovery effect. This study is divided into five parts. The research method of the situational experiment will be used to collect research data, explore the impact of empathy on consumer forgiveness and the impact of consumer forgiveness on consumer repurchase intention, and test the moderating effect of online store reputation on the impact of consumer forgiveness on consumer repurchase intention.

This study will have important theoretical and practical significance. In terms of theoretical significance, this study will deepen the research on the relationship between empathy and consumer forgiveness, expand its theoretical application scenarios and research vision, and enrich the online shopping service recovery theory. This study will also expand the applicable scenarios of online store reputation and enrich the connotation of online store reputation and service recovery research. In terms of practical significance, this study is not only conducive for online stores to improve online shopping consumers’ willingness to forgive after service failure, but is also conducive for online stores to improve their own reputation level, so as to provide a service recovery effect and promote the healthy development of online shopping business models.

## Literature Review and Research Hypothesis

### Service Failure and Service Recovery

Service failure is a service that does not meet the expectations of consumers; it is the contact environment that causes consumers’ dissatisfaction (Peng and Jing, 2005). Service failure includes result failure and process failure. Result failure occurs when the service provider fails to achieve the basic service content and meet customer expectations, while process failure mainly refers to the unpleasant service experience in terms of delivery mode (Bitner et al., 1990).

Service recovery is the response and remedial measures taken by service providers against consumers’ dissatisfaction and complaints after service failure (Kelly, 2018). Both offline and online consumers will feel that the resources exchanged have depreciated when they encounter service failure, resulting in psychological dissatisfaction and complaints. Therefore, service recovery is needed to eliminate the negative impact of service failure (Crisafulli and Singh, 2017). The service recovery strategy is the focus of current service failure and service recovery research. Scholars believe that in service recovery, service providers need to confirm, evaluate, explain, apologize, and compensate for service failure to eliminate consumers’ dissatisfaction (Boshof, 1999). Heejung (2012) proposed that in service recovery, different service recovery strategies should be adopted in combination with the individual characteristics of consumers to make service recovery more targeted and effective. Assefa (2014) showed through empirical research that procedural fairness, interactive fairness, and distribution fairness are positively correlated with service recovery satisfaction and service recovery satisfaction is also positively correlated with customer loyalty. Siu et al. (2014) confirmed through investigation and research that good CSR cognition helps to reduce the negative impact of internal cause attribution on customer identification and ultimately helps to improve service recovery satisfaction. In tourism service recovery, the verbal and non-verbal behaviors of frontline employees have a significant impact on service recovery cooperation willingness (Huang, 2021). Yang et al. (2020) confirmed through research that high customer participation in service failure and service recovery satisfaction can be effectively improved when an “immediate recovery opportunity” is matched and low customer participation service failure is matched with a “preventive recovery opportunity.” Ahmad et al. (2021) confirmed that there is a positive and significant relationship between service sales dexterity and service recovery performance and adaptive sales behavior, while there is a significant relationship between adaptive sales behavior and service recovery performance. Experimental research of Liu et al. (2021) confirmed that taste perception can activate relevant conceptual metaphors, resulting in differences in service recovery satisfaction.

For the research on the online shopping service recovery strategy, Baliga et al. (2021) focused on the service recovery barriers in the B2B market, identified the service recovery barriers in the B2B market, and explored how to identify the key driving barriers. Das et al. (2019) believed that retailers and service providers need to improve the store environment and formulate successful complaint management strategies to improve service recovery satisfaction. Wang and Lu (2018) confirmed that there is a significant positive relationship between service remedies and perceived fairness and there is a significant positive relationship between perceived fairness and customer satisfaction. An e-tailer’s watchfulness toward the strengthening of its online ethics synergistically redeems recovery satisfaction (Costers et al., 2019; Roy et al., 2022). The research of Quan and Li (2021) shows that in service recovery, brand trust, brand intimacy, and empathy have a significant positive impact on consumer forgiveness and consumer forgiveness plays a significant intermediary effect in the influence path of attribution, severity, brand trust, brand intimacy, and empathy on repurchase intention. However, the current literature still lacks research on empathy and consumer forgiveness in the context of online shopping services and consumer repurchase intention, so it needs to be further discussed.

### Empathy and Consumer Forgiveness

Stimulus organization response (S-O-R) theory points out that the external environment will affect individual cognition and emotion as a stimulus and the stimulated cognition and emotion will eventually affect individual behavior (Song et al., 2019; Chen et al., 2020). Stimulation is a kind of external influence, which can affect people’s psychological state and urge people to respond. Stimulation affects psychology through the consciousness of the recipient. The recipient is an organism. After being stimulated, it forms a conscious or unconscious psychological response of the organism (Jacoby, 2002; Meilhan, 2019). In the current research on consumer cognition, consumer perception, and consumer behavior intention, many scholars utilize S-O-R theory and produce many research results (Escalas, 2004; Graessley et al., 2019; Rydell and Kucera, 2021; Watson and Popescu, 2021). S-O-R theory is also suitable for analysis in this study. After the failure of online store service, the service recovery given by the online store to the consumer is to stimulate the consumer and cause empathy tendency, so as to promote the formation of consumer forgiveness and consumer repurchase intention.

Empathy means that an individual can correctly perceive the current emotions of others. It can stimulate individuals’ altruistic motivation and trigger their prosocial behavior. Its goal is to relieve others’ difficulties and increase others’ welfare (Batson, 1991; Hoffman, 1994). In the study of consumer behavior, empathy refers to consumers’ emotional conscious and unconscious integration and identification with external stimuli, which is manifested in the absorption of emotional responses to external stimuli (Escalas and Stern, 2003). Wang and Li (2016) believe that an individual’s moral way of thinking will be affected by empathy, but empathy is not the only factor that affects moral thinking.

Enright et al. (1992) hold that forgiveness refers to the willingness and behavior of the victim to understand, be kind, and accept the offender after being hurt by the offender. Consumer forgiveness refers to the psychological change process in which consumers choose to be considerate and tolerant of the merchant’s mistakes or offensive behaviors by releasing their negative emotions after experiencing the mistakes of online shopping services, so as to increase the willingness to rebuild relationships (Ho, 2020; Huang and Chang, 2020). The influencing factors of consumer forgiveness include individual cognitive factors, emotional factors, relationship quality factors, and situational factors (Sun, 2012; Ran et al., 2016). After the crisis, the sincere personification strategy can better improve consumers’ willingness to forgive than the ability brand personification strategy, and when consumers have high loyalty to the crisis brand, the influence difference of the brand personification strategy on consumers’ willingness to forgive will be weakened (Sun and Guo, 2019; Zeng, 2021). Babin et al. (2021) conducted an empirical study on older American consumers (aged 55 years and above) and confirmed that the sense of fairness affects behavioral forgiveness through emotional forgiveness. Bast and Barnes (2015) conducted a cross-situational and cross-cultural empirical study and believed that when people tend to empathize with each other, they are more likely to forgive each other’s offensive behavior to a certain extent. In the current research on service recovery, Wei et al. (2020) confirmed that emotional recovery can more effectively stimulate consumers’ sympathy and forgiveness for service providers than economic recovery. In the case of service failure, effective service recovery strategies can guide the generation of empathy. Consumers driven by empathy consciously reduce negative motives such as alienation and revenge and enhance tolerance and kindness (Mccullough et al., 1998). Sun and Sun (2021) followed the research logic of attribution theory and S-O-R theory and confirmed through empirical research that consumer empathy plays an intermediary effect in the impact of service recovery strategies on consumers’ willingness to forgive. In the online shopping service recovery scenario, when consumers empathize with the online store, they will be able to understand the reasons for the service failure, so as to forgive their behavior to a certain extent. Therefore, the following research hypotheses are proposed:

H1: Empathy has a significant positive impact on consumer forgiveness.

### Consumer Repurchase Intention and Online Store Reputation

Consumer purchase intention refers to the tendency of consumers to purchase a product or service (Qi and Zhang, 2021). Consumer repurchase intention is the tendency of consumers to decide to continue to buy products or services of an enterprise in the future, which generally includes two dimensions: repeated purchase and willingness to recommend (Zeithaml, 1996). When consumers have a sense of belonging or dependence on the enterprise’s products to a certain extent, they will psychologically promise to continue to buy the enterprise’s products in a certain period of time in the future, resulting in the tendency of repurchase intention (Weng et al., 2016). Empirical research by Wang and Li (2020) confirmed that consumer forgiveness in online shopping has a positive impact on consumers’ willingness to repurchase. In the field of service recovery, based on the service recovery scenario, some scholars have confirmed that consumer forgiveness has a significant positive impact on sustained trust (Huang and Chang, 2020). It is also confirmed that consumer forgiveness has a significant mediating effect on the influence path of attribution, severity, brand trust, brand intimacy, and empathy on repurchase intention (Hua, 2020; Quan and Li, 2021). In the online shopping service recovery scenario, when consumers forgive the service failure of the online store, it will improve their repurchase intention. In addition, empathy also has an indirect impact on consumer forgiveness. According to the above literature analysis, this study puts forward the following research hypotheses:

H2: Consumer forgiveness has a significant positive impact on consumer repurchase intention.

H3: Consumer forgiveness has a mediating effect between empathy and consumer repurchase intention.

Reputation can be divided into individual reputation and collective reputation. Collective reputation is a collection of individual reputations, but individual reputation will have an impact on collective reputation, including a positive influence and negative influence (Tirole, 1996; Liu et al., 2014). Current research believes that enterprises with high reputation will be able to obtain higher income, so as to attract better employees, reduce costs, and obtain price advantages (Huang and Sun, 2016). Shopping websites are composed of a large number of online stores, which only provide a trading platform and services. The actual transactions are between online stores and buyers. Therefore, website reputation is collective reputation, while online store reputation is individual reputation. Empirical studies have confirmed that the establishment of online platform reputation can encourage online shopping platforms to fulfill their psychological obligations, contract responsibility, and maintain long-term psychological contract relationships and reputation (Li and Zhou, 2017). The reputation of online shopping platforms perceived by consumers has a positive impact on the use level of reputation information and online shopping amount (Lin, 2016). Li (2016), through the statistical analysis of the models of online store reputation, online store performance, and relationship quality by collecting the transaction data of Taobao in China, showed that online store reputation has a significant positive impact on online store performance and relationship quality has a moderating effect on the impact mechanism of online store reputation on online store performance. In the scenario of online shopping service recovery, if the online store has a good reputation, it will strengthen the relationship between consumer forgiveness and consumer repurchase intention and have a positive moderating effect. Therefore, this study puts forward the following research hypothesis:

H4: Online store reputation has a positive moderating effect on the impact of consumer forgiveness on consumer repurchase intention.

**FIGURE 1:**
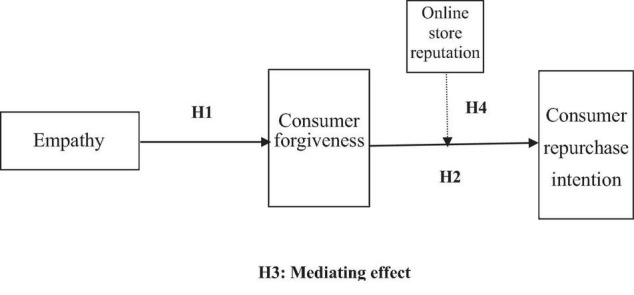
Research model.

Based on the theoretical basis of empathy, consumer forgiveness, online store reputation, and consumer repurchase intention, combined with the online shopping service recovery scenario and based on the above literature research and research hypothesis analysis, this study establishes the research model of this study, as shown in Figure 1.

### Research Design

#### Experimental Design

Although many online shopping service failure events occur everyday, due to the limitation of time and space, it is still difficult for researchers to collect sample data at the scene of service failure and service recovery. Therefore, in order to improve the scientificity and effectiveness of online shopping service recovery research, this study refers to Zhang et al. (2013), Chen et al. (2017), and Guan et al. (2017) who decided to use the method of a scenario experiment to carry out empirical research on the basis of referring to real online shopping service failure cases. In order to ensure the external validity and pertinence of the experiment, the experimental materials came from online shopping service failure and service recovery events reported by Chinese media and were appropriately adapted according to the research needs.

In the scenario experiment, the researcher designed a service failure event when consumers buy disinfection cabinets and the online store took service recovery. There are four experimental scenarios: High empathy vs. high consumer forgiveness vs. high online store reputation, high empathy vs. high consumer forgiveness vs. low online store reputation, low empathy vs. low consumer forgiveness vs. high online store reputation, and low empathy vs. low consumer forgiveness vs. low online store reputation. In the scenario experiment, all the subjects were randomly divided into the four groups and each group was set one scenario. The four scenarios are described below.

##### Scenario 1

In the process of selling refrigerators, the online store failed due to quality reasons and the online store took the initiative to take service recovery measures. Consumers have a high tendency of empathy, feel that service failure is inevitable, and gradually have a willingness to understand and forgive the online store. Moreover, the online store has a high reputation and consumers will continue to buy the goods of the online store.

##### Scenario 2

In the process of selling refrigerators, the online store failed due to quality reasons, and the online store took the initiative to take service recovery measures. Consumers have a high tendency of empathy and feel that service failure is inevitable, so they gradually have a willingness to understand and forgive the online store. However, the reputation of the online store is relatively low, which reduces consumers’ willingness to repurchase.

##### Scenario 3

In the process of selling refrigerators, the online store failed due to quality reasons, and the online store took the initiative to take service recovery measures. Consumers have a relatively low tendency of empathy. They think that if they operate online stores, they will avoid these problems. Consumers think it is incomprehensible and unforgivable. The online store has a high reputation, which is conducive to improving consumer repurchase intention.

##### Scenario 4

In the process of selling refrigerators, the online store failed due to quality reasons, and the online store took the initiative to take service recovery measures. Consumers have a relatively low tendency of empathy. They think that if they operate online stores, they will avoid these problems. Consumers think it is incomprehensible and unforgivable. At the same time, the online store has a low reputation, and consumers will no longer buy the goods of the online store.

### Variable Measurement

This study has four measurement variables: empathy, consumer forgiveness, online store reputation, and consumer repurchase intention. These four measurement variables constitute the questionnaire of the scenario experiment. In order to ensure that the measurement items of the four variables comply with the characteristics of the online shopping service recovery scenario and make the measurement more scientific and targeted, this study adopts the relevant scales of authoritative literature in their respective fields and modifies them accordingly as per the online shopping service recovery scenario.

The measurement items of empathy in this study refer to the scale of Mccullough et al. (1998) and five items are set. The measurement items of consumer forgiveness refer to the scale development results of Finkel et al. (2002) and Wang and Li (2020), and four items are set. The online store reputation measurement items refer to the research results of Ponzi et al. (2011) and Wang and Guo (2018), four measurement items are set. The measurement items of consumer repurchase intention in this study refer to the scale development results of Zeithaml (2000) and Xu et al. (2018) and five measurement items are set. The 18 measurement items in this study are measured by a Likert 5-point scale and the five options include “very inconsistent, inconsistent, basically consistent, consistent, and very consistent,” which are scored as “1, 2, 3, 4, 5,” respectively. Therefore, the questionnaire of the scenario experiment consists of 4 variables and 18 measurement items, which will be investigated for the subjects in the scenario experiment.

### Scenario Experiment Implementation

From August to September 2021, this study conducted scenario experiments on citizens in Guilin, Nanning, and Liuzhou, as well as farmers in Yangshuo County, China. Three researchers, eleven college student volunteers, and eleven community and village committee staff participated in the organization and implementation of the scenario experiment. This study mainly collected subjects through the following two ways: First, we cooperated with urban communities (grassroots management organizations in Chinese cities) and community workers who collected qualified subjects through community social platforms (QQ group and WeChat group); second, we cooperated with Chinese village committees (China’s rural grassroots management organizations) and the staff of the village committee who solicited qualified subjects through the social platform (QQ group) of the village committee. All the above subjects were required to be over 18 years old, have online shopping experience, and participate voluntarily.

In the scenario experiment, the researcher explained the four scenarios and asked the subjects to choose one of the scenarios, carefully read the text description of their scenario, and put the individual under the scenario. In order to improve the effectiveness of the scenario experiment and the quality of questionnaire filling, 3 researchers and 11 college student volunteers answered the experimental operation process and questionnaire filling methods in detail, which improved the accuracy of questionnaire filling and avoided the problem of non-response bias.

In the scenario experiment, we distributed 273 questionnaires and recovered 264. Excluding incomplete questionnaires and questionnaires with inconsistent answers, there were 247 valid questionnaires. The sample distribution is given in Table 1.

**TABLE 1:** Sample statistics.

One class indicators	Two class indicators	Sample size	Percentage	One class indicators	Two class indicators	Sample size	Percentage
Gender	Male	121	48.98%	Education	Middle and primary school	94	38.06%
	Female	126	51.02%		College degree	68	27.53%
Age	18–25 years	53	21.46%		Bachelor	53	21.46%
	26–35 years	64	25.91%		Master	24	9.72%
	36–45 years	68	27.53%		Doctor	8	3.24%
	46–59 years	43	17.41%	Occupation	Civil servant	18	7.29%
	Over 60 years	19	7.69%		Professional	53	21.46%
Online shopping years	Under 1 years	50	20.24%		Enterprise staff	64	25.91%
	1–3 years	77	31.17%		Farmer	58	23.48%
	4–5 years	64	25.91%		Student	39	15.79%
	Over 5 years	56	22.67%		Other	15	6.07%

### Data Analysis

#### Common Method Bias Test

Common method variance (CMV) suggests that using the same measurement tool will lead to false common variation among traits, which is common in data measured by a self-report scale (Xiong et al., 2013). The deviation caused by CMV is called common method bias (CMB), which is a systematic error independent of traits that affects the validity of measurement (Podsakoff et al., 2003; Richardson et al., 2009). Although the measurement questions of the four variables in this study refer to the authoritative scale and are measured by a Likert 5-point scale, they all belong to the scope of a self-report scale. Therefore, there may be CMB in the situational use data of this study, which needs to be tested for CMB.

The Harman single factor method often uses exploratory factor analysis (EFA) to test CMB. The EFA method suggests that there is a method factor to explain the common variation of all items with different characteristics in a study. The more variation explained by the factor, the more serious the deviation (Podsakoff et al., 2003; Wang, 2020). It is generally believed that if the single factor interpretation variation obtained by EFA (no rotation) does not exceed 40%, CMB is not serious (Tang and Wen, 2020). This study refers to Podsakoff et al. (2003). Tang and Wen (2020) proposed the Harman single factor test method to test common method deviation. The research results show that in the test of common method deviation, 18 common factors are analyzed and the variance interpretation percentage of the first common factor is 21.95%, which is significantly less than 40%. Therefore, it can be considered that there is no serious common method deviation in this study.

#### Reliability and Validity Test

In the scenario experiment, this study uses a questionnaire to collect experimental data. The Cronbach’s α is used for the reliability of this questionnaire; when Cronbach’s α is greater than 0.70, it indicates that the variable passes the reliability test (Hair and Black, 2006). As shown in Table 2, Cronbach’s four variables of α are between 0.732 and 0.829, both are greater than 0.70, which indicates that the reliability of the questionnaire has passed the test.

**TABLE 2:** Test for reliability, convergent, and construct validity.

Variables	Item	Normalized Load factor	*T* Value	Cronbach’s α	CR	AVE
Empathy	(1) I understand the reasons for mistakes in online stores	0.823	6.133			
	(2) I can understand the difficulties of online stores	0.768	4.043			
	(3) I would imagine what would happen if it were an online store	0.674	3.224	0.812	0.877	0.589
	(4) The service failure of the online store is just a case	0.801	6.776			
	(5) Online stores are thinking about consumers	0.764	2.904			
Consumer forgiveness	(6) I agree with the service recovery of the online store	0.819	5.533			
	(7) I agree with the material compensation of the online store	0.711	4.166			
	(8) I agree with the online store’s apology	0.702	4.427	0.732	0.850	0.588
	(9) I forgive the mistakes of the online store	0.826	6.781			
Online store reputation	(10) The online evaluation of the online store is very high	0.635	2.272			
	(11) People around me have recommended this online store to me	0.714	4.305	0.746	0.814	0.524
	(12) The online store has a good reputation	0.738	4.795			
	(13) There are few negative news from online stores	0.799	6.167			
Consumer repurchase intention	(14) I am satisfied with the service of the online store	0.819	4.081			
	(15) I will continue to buy the goods of the online store	0.857	5.433			
	(16) I will recommend the online store to the people around me	0.794	3.768	0.829	0.915	0.682
	(17) I will trust the online store more	0.877	6.726			
	(18) I will become a loyal consumer of the online store	0.779	4.265			

This study also tested the validity of the questionnaire. In terms of content validity, all the items of the questionnaire refer to the representative research results in relevant research fields and are modified in combination with the online store service recovery situation, indicating that the questionnaire has good content validity. The convergence validity is shown in Table 2. The standardized load factor of 18 items is greater than 0.50 and the *T* value is greater than 1.96. The combined reliability (CR) values of the four variables are greater than 0.70 and average variance extracted (AVE) is greater than 0.50, which meets the test criteria of convergence validity (Hair and Black, 2006; Wu, 2010). Therefore, the questionnaire passed the convergence validity test.

This study also conducted a discriminant validity test and constructive validity test. As shown in Table 3, the square root of the AVE of the four variables is greater than the correlation coefficient between the variable and other variables. According to the standard of Wu (2010), the questionnaire has good differential validity. In the construct validity test, which used AMOS22.0 for confirmatory factor analysis (CFA), the results of the confirmatory factor analysis of the overall fit of the model are shown in Table 4. The criteria for a good model are: χ^2^/DF should be less than 5, comparative fit index (CFI) and Tucker–Lewis index (TLI) should be greater than 0.90, standardized root mean squared residual (SRMR) should be less than 0.05, and root mean square error of approximation (RMSEA) should be less than 0.1 (Wu, 2010). Therefore, the model of this study reached the standard of a good model and the questionnaire passed the test of constructive validity.

**TABLE 3:** Test for discriminant validity.

Variables	1	2	3	4
(1) Empathy	0.767			
(2) Consumer forgiveness	0.616	0.766		
(3) Online store reputation	0.101	0.094	0.724	
(4) Consumer repurchase intention	0.496	0.668	0.622	0.826

*The value on the diagonal is the square root of AVE, and other data are the correlation coefficients between corresponding variables.*

**TABLE 4:** Research model fit.

Fit index	χ ^2^	DF	χ ^2^/DF	CFI	TLI	SRMR	RMSEA
Index value	317.043	149	2.128	0.941	0.944	0.035	0.053

#### Test for Direct Impact Relationship

This study uses SPSS version 22.0 software for multilevel regression analysis to test the direct influence relationship and moderating effect between variables. The variance expansion factor (VIF) of this study is between 2.462 and 3.998. The VIF is lower than the empirical value of 10, which proves that there is no multicollinearity problem in this study model (Li et al., 2019).

In order to verify the direct impact of empathy on consumer forgiveness, this study constructed model 1 and model 2. As shown in Table 5, model 1 is the regression of control variables to consumer forgiveness. The results show that the influence between each control variable and consumer forgiveness is not statistically significant. Model 2 shows that empathy has a significant positive impact on consumer forgiveness (β = 0.659, *P* < 0.01), so H1 passed the test.

**TABLE 5:** Multilevel regression analysis.

Variable	Consumer forgiveness		Consumer repurchase intention		
	Model 1	Model 2	Model 3	Model 4	Model 5
Intercept	3.004*	2.839*	2.698*	3.371**	3.726**
**Control** **variables**					
Gender	0.111	–0.052	0.109	0.103	0.098
Age	0.119	–0.097	–0.136	–0.159	–0.127
Online shopping year	0.083	0.113	0.124	0.141	0.079
Education	0.127	0.129	0.138	0.115	0.119
Occupation	0.023	0.037	–0.137	0.099	0.034
**Independent** **variable**					
Empathy		0.659**			
**Consumer forgiveness**				0.626**	
**Moderating** **variables**					
Online store reputation					0.214*
**Interaction** **Item**					
Consumer forgiveness × Online store reputation					0.341*
***R* ^2^**	0.061	0.307	0.139	0.534	0.421
Δ***R*^2^**		0.071		0.068	0.033
***F***	2.984*	3.899**	4.976***	3.409**	4.436**

*p < 0.05, **p < 0.01, ***p < 0.001.

The direct impact of consumer forgiveness on consumer repurchase intention is tested by constructing model 3 and model 4. First, the control variables are included in model 3. The influence of each control variable on consumer repurchase intention is not statistically significant. Second, incorporating consumer forgiveness into model 5, the results show that consumer forgiveness has a significant positive impact on consumer repurchase intention (β = 0.626, *P* < 0.01), so H2 passed the test.

#### Moderating Effect Test

This study examines the moderating effect of online store reputation on consumer forgiveness and consumer repurchase intention. As shown in model 5, from the multilevel regression results, it is found that the interaction coefficient between consumer forgiveness and online store reputation is positive and statistically significant (β = 0.341, *P* < 0.05), indicating that online store reputation has a positive moderating effect on the relationship between consumer forgiveness and consumer repurchase intention, and H4 passed the test.

**FIGURE 2:**
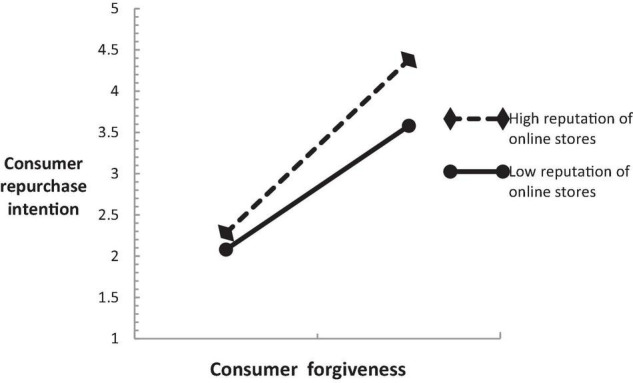
Moderating effect diagram.

In order to better show the moderating effect of online store reputation between consumer forgiveness and consumer repurchase intention, this study draws a moderating effect diagram, as shown in Figure 2. When the online stores reputation is low, consumer forgiveness has a weak positive impact on consumer repurchase intention (β = 0.366, *P* < 0.01), and when the online store reputation is high, consumer forgiveness has a strong positive impact on consumer repurchase intention (β = 0.763,*P* < 0.01). Therefore, the online store reputation has improved the positive impact of consumer forgiveness on consumer repurchase intention, with a positive regulatory effect, and H4 has been further verified.

#### Mediating Effect Test

For the simple mediation effect test, the bootstrap method has obvious advantages and will be more scientific and accurate than the causal stepwise regression method (Chen et al., 2013). The mediating effect of this study is the simple mediating effect, and the bootstrap method is also suitable for the mediating test. Therefore, we used the Process Ver 3.5 plug-in released by Hayes in May 2020 to test the mediation effect.

According to the suggestions of Hayes and Scharkow (2013) and Wen and Ye (2014), the specific operation methods of the mediating effect test in this study are as follows: First, download the Process version 3.5 plug-in on the official website, and then install it as a plug-in in SPSS version 22.0. In SPSS version 22.0, first select “analyze regression process” to enter the operation and change the operation variables (independent variable, intermediate variable, and dependent variable), select the option box, set the model serial number and sampling times to 4 and 5,000, respectively, select “BIA corrected” as the sampling method, set the 95% confidence interval, and finally click “OK” to run the intermediate effect test.

The mediating effect path of this study is “Empathy → Consumer forgiveness → Consumer repurchase intention.” The mediating effect test results are shown in Table 6. The indirect effect value of the mediating path is 0.264, accounting for 38.77% of the total effect, indicating that consumer forgiveness has a mediating effect between empathy and consumer repurchase intention, H3 passed the test.

**TABLE 6:** Test results of mediating effect.

Mediating effect path	Indirect effect value	Standard error	Upper limit	Lower limit	Effect proportion
Empathy → Consumer forgiveness → Consumer repurchase intention	0.264	0.009	0.102	0.452	38.77%
Total effect	0.681	0.012	0.387	0.785	100%

## Conclusion and Discussion

### Research Conclusion

First, the results of this study show that empathy has a significant positive impact on consumer forgiveness in the context of online shopping service recovery (β = 0.659, *P* < 0.01), H1 passed the test. Why the above research results? We believe that in online shopping service recovery, if consumers have a tendency to empathize with the online store where the service failure occurs, they will think more from the standpoint of the online store. For example, consumers will think that the service failure is inevitable, and doing anything may cause mistakes, so they cannot blame the online store too much, and so on. Therefore, in the online shopping service recovery scenario, consumers’ empathy tendency will positively affect consumer forgiveness.

Second, this study confirms that consumer forgiveness has a significant positive impact on consumer repurchase intention in online shopping service recovery (β = 0.626, *P* < 0.01), H2 passed the test. In addition, consumer forgiveness has a mediating effect between empathy and consumer repurchase intention (the value is 0.264), so H3 also passed the test. The above research results show that in online shopping service recovery, if consumers forgive the service failure of the online store, and consumers’ psychological complaints and negative comments about the online store have been reduced or eliminated, consumers’ willingness to repurchase will be improved.

Third, from the multilevel regression results, this study found that the interaction coefficient between consumer forgiveness and online store reputation is positive and statistically significant (β = 0.341, *P* < 0.05). The study confirmed that when the reputation of online stores is low, consumer forgiveness has a weak positive impact on consumer repurchase intention (β = 0.366, *P* < 0.01), and when the online store reputation is high, consumer forgiveness has a strong positive impact on consumer repurchase intention (β = 0.763, *P* < 0.01). The above research results show that online store reputation has a positive moderating effect on the relationship between consumer forgiveness and consumer repurchase intention, and H4 passes the test. Therefore, consumer forgiveness has a positive impact on consumer repurchase intention, and online store reputation will strengthen the positive relationship between the two. In service recovery, if the online store has a high reputation, good reputation, and good online comments, it will be easier to form consumer repurchase intention. High online store reputation is the “catalyst” to promote consumer repurchase intention. On the contrary, low online store reputation will weaken the positive impact of consumer forgiveness on consumer repurchase intention.

### Theoretical Contributions

First, this study confirms that empathy has a significant positive impact on consumer forgiveness in the context of online shopping service recovery. The above research conclusions confirm the existing research that consumers driven by empathy will enhance positive motives such as tolerance and kindness, so as to help repair the damaged relationship (Mccullough et al., 1998). However, there are few existing studies on the impact of empathy on consumer forgiveness in the context of online shopping service recovery. Therefore, this study expands the research vision of empathy and consumer forgiveness, deepens the research on the relationship between empathy and consumer forgiveness, and will enrich the theoretical research on online shopping service recovery.

Second, this study confirms that in the online shopping service recovery scenario, consumer forgiveness can promote consumer repurchase intention, and consumer forgiveness plays a mediating role between empathy and consumer repurchase intention. However, there are few studies on the relationship between empathy, consumer forgiveness, and consumer repurchase intention in the context of online shopping service recovery. The existing research is mainly based on other research scenarios, and rarely brings empathy into the research framework, such as Weng et al. (2016) and Quan and Li (2021), etc. Therefore, this research realizes the innovation of research scenarios, extends the research scenarios to the field of online shopping service recovery, and expands the research framework and research vision.

Third, the results of this study show that in the online shopping service recovery scenario, online store reputation has a positive moderating effect on the impact of consumer forgiveness on consumer repurchase intention. In this study, some scholars believe that consumer-perceived online shopping platform reputation has a positive impact on the use level of reputation information and online shopping amount (Lin, 2016). Others believe that online store reputation has a significant positive impact on online store performance, and relationship quality has a moderating effect on the impact mechanism of online store reputation on online store performance (Li, 2016). However, the existing studies rarely involve online shopping service recovery scenarios, and there are few articles that study online store reputation as a moderating variable. This study takes online store reputation as a moderating variable to study the impact mechanism of online shopping service recovery, which will expand the applicable scenarios of online store reputation. In addition, this study is also conducive to better explain the role of online store reputation in service recovery and enrich the research connotation of online store reputation and service recovery.

### Practical Applications

First, after service failure, online stores should pay more attention to creating conditions to promote consumers’ empathy. In service recovery, online stores should show consumers the difficulty of service work and the inevitability of service failure. For example, through communication with consumers, they should inform them of the difficulty of achieving perfect management in logistics and product quality control in current online shopping services, and compare them with other businesses and other industries to make consumers sympathize with the service work of online stores and look at problems from the perspective of online stores. For impatient consumers, online stores also need to sincerely apologize, respect, comfort, emotional counsel, and fairly treat their customers, so as to reduce consumers’ negative emotions and enhance consumers’ empathy tendency.

Second, online stores should improve consumers’ willingness to forgive through various ways. This study shows that consumer forgiveness can promote the formation of consumer repurchase intention, and consumer forgiveness plays a mediating role between empathy and consumer repurchase intention. Therefore, after service failure, the online store needs to carry out targeted service recovery training for employees to improve their service recovery ability. On the basis of consumers’ empathy, online stores should further guide consumers to forgive service failure. Online stores should focus on resolving the complaints and anger of some consumers after service failure, prevent the tendency of emotional extremes, and improve the willingness of online shopping consumers to forgive.

Third, online store reputation is the foundation of survival. They must do everything possible to improve online store reputation. The results of this study show that online store reputation has a positive moderating effect on the impact of consumer forgiveness on consumer repurchase intention. How to improve the reputation of online stores? This is a problem that online stores should often think about. In operation and management, online stores should improve product quality and establish the concept of “quality wins.” Unqualified products will not be sold. In online shopping services, online stores should strictly abide by relevant laws and regulations, eliminate illegal business practices, be honest and trustworthy, and establish a good public image. Online stores can also use hot events and online popularity to carry out public relations activities to improve the attention rate and online store reputation.

### Research Limitations and Prospects

First, in terms of sample sources, this study obtained the research samples through the scenario experiment. The samples mainly came from the citizens of Guilin, Nanning, and Liuzhou, as well as farmers in Yangshuo County, China, but lack samples of other cities and rural areas in China. Therefore, in this study, we should expand the sample source and increase the samples of other cities and rural areas in China. In the comparative study of consumer culture, samples from other countries should be added.

Second, in terms of research methods, the main research method of this study was the scenario experiment. The scenario experiment method has the advantages of good controllability, low cost, and no ethical problems. However, the problems dealt with in the scenario experiment are abstract and narrow, which are only limited to the scenario described in the scenario experiment, and there may be control variables, while the real service recovery scenario is not necessarily similar to the service recovery scenario described in the scenario experiment. Therefore, the research conclusions obtained through scenario experiments may have limited explanatory power to practical problems. Based on the limitations of the scenario experiment method, in future research, we should combine a variety of research methods to conduct online shopping service recovery research, such as the web crawler method, interview method, and case analysis method, so as to make the research conclusion more scientific and effective. For example, the web crawler system is developed through Python software, so as to obtain the times consumers visit an online store, the length of stay in the online store, the products they pay attention to, the purchase amount and times, etc., so as to make up for the deficiency of the scenario experiment method.

Third, in terms of research content, this study explored the relationship between empathy and consumer forgiveness on consumer repurchase intention, and tested the regulatory effect of online store reputation, but did not consider broader issues, such as consumers’ face, perceived value, and negative emotions, which may have an important impact on the effect of service recovery. Therefore, in future research on online shopping service recovery, we need to expand the research framework and optimize the research model to better explain the practical problems in online shopping service recovery.

## Data Availability Statement

The original contributions presented in the study are included in the article/supplementary material, further inquiries can be directed to the corresponding author/s.

## Ethics Statement

The studies involving human participants were reviewed and approved by Academic Department of Guilin University of Technology, China. Written informed consent for participation was not required for this study in accordance with the national legislation and the institutional requirements.

## Author Contributions

JW was responsible for proposing research concepts, research hypotheses, scenario experiments and data analysis, and wrote and revised the manuscript. YM and ZH participated in the scenario experiment and manuscript revision. YM and ZW participated in data analysis. All authors contributed to the article and approved the submitted version.

## Conflict of Interest

The authors declare that the research was conducted in the absence of any commercial or financial relationships that could be construed as a potential conflict of interest.

## Publisher’s Note

All claims expressed in this article are solely those of the authors and do not necessarily represent those of their affiliated organizations, or those of the publisher, the editors and the reviewers. Any product that may be evaluated in this article, or claim that may be made by its manufacturer, is not guaranteed or endorsed by the publisher.
